# Clinical governance network for clinical audit to improve quality in epithelial ovarian cancer management

**DOI:** 10.1186/1757-2215-6-19

**Published:** 2013-03-31

**Authors:** Vincenzo Dario Mandato, Martino Abrate, Pierandrea De Iaco, Debora Pirillo, Gino Ciarlini, Maurizio Leoni, Giuseppe Comerci, Alessandro Ventura, Bruno Lenzi, Andrea Amadori, Federica Rosati, Ruby Martinello, Rossana De Palma, Chiara Ventura, Laura Maria Beatrice Belotti, Debora Formisano, Giovanni Battista La Sala

**Affiliations:** 1 Department of Obstetrics and Gynecology, Arcispedale S. Maria Nuova- IRCCS, Reggio Emilia, Italy; 2 Department of Obstetrics and Gynecology, University Hospital S. Orsola Malpighi, Bologna, Italy; 3 Oncology and Gynecology Unit, Ospedale Civile, Ravenna, Italy; 4 Obstetrics and Gynecology Unit Ospedale Civile, Ravenna, Italy; 5 Department of Obstetrics and Gynecology, Ospedale Civile, Guastalla, Reggio Emilia, Italy; 6 Unit of Medicine, Hospital of Argenta, Ferrara, Italy; 7 Unit of Obstetrics and Gynecology, Ospedale G.B. Morgagni-L.Pierantoni, Forlì, Italy; 8 Unit of Obstetrics and Gynecology, Ospedale degli Infermi, Rimini, Italy; 9 Unit of Obstetrics and Gynecology, University Hospital S. Anna, Ferrara, Italy; 10 Clinical Area Government, Health and Social Agency of Emilia-Romagna, Emilia-Romagna, Italy; 11 Statistics and Clinical Epidemiology Unit, Arcispedale Santa Maria Nuova - IRCCS, Reggio Emilia, Italy; 12 Department of Obstetrics and Gynecology, Arcispedale S. Maria Nuova- IRCCS; University of Modena and Reggio Emilia, Reggio Emilia, Italy

**Keywords:** Epithelial ovarian cancer, Centralized care, Clinical audit, Quality of care, Guide line

## Abstract

**Background:**

Epithelial ovarian cancer (EOC) is the most lethal gynecological cancer. Several hospitals throughout the region provide primary treatment for these patients and it is well know that treatment quality is correlated to the hospital that delivers. The aim of this study was to investigate the management and treatment of EOC in a Region of the North Italy (Emilia-Romagna, Italy).

**Methods:**

A multidisciplinary group made up of 11 physicians and 3 biostatisticians was formed in 2009 to perform clinical audits in order to identify quality indicators and to develop Region-wide workup in accordance with the principles of evidence-based medicine (EBM). The rationale was that, by setting up an oncogynecology network so as to achieve the best clinical practice, critical points would decrease or even be eliminated. Analysis of cases was based on the review of the medical records.

**Results:**

614 EOC patients treated between 2007 and 2008 were identified. We found only 2 high-volume hospitals (≥ 21 patients/year), 3 medium-volume hospitals (11–20 operated patients/year), and 7 low-volume hospitals (≤ 10 operated patients /year). Only 222 patients (76.3%) had a histological diagnosis, FIGO surgical staging was reported only in 206 patients (70.9%) but not all standard surgical procedures were always performed, residual disease were not reported in all patients. No standard number of neoadjuvant chemotherapy cycles was observed.

**Conclusions:**

The differences in terms of treatments provided led the multidisciplinary group to identify reference centers, to promote centralization, to ensure uniform and adequate treatment to patients treated in regional centers and to promote a new audit involving all regional hospitals to a complete review of the all the EOC patients.

## Background

Epithelial ovarian cancer (EOC) is the most lethal gynecological cancer. Worldwide, there are 224,747 new cases yearly and an estimated 140,163 disease-related deaths [1]. In Europe, approximately 66,700 new ovarian cancer cases are diagnosed yearly, with the highest incidence in the Northern European countries and the United Kingdom [2,3]. The lifetime risk varies from 1.1 to 1.6 in Europe and the United States (US) [4]. The majority of women (about 65%) are diagnosed at advanced International Federation of Obstetricians and Gynecologists (FIGO) stage (III-IV) 5-year survival is only 48.8% of all the patients, and the median overall survival is 24 months. However, the 5-year-survival rate varies widely among European countries, from 25.6% to 51.4% [5,6].

In the Emilia-Romagna Region, a region of the North Italy, there is a yearly average of 17.6 new EOC per 100,000 females (about 401 new cases); 40 hospitals throughout the region provide primary treatment for these patients. The 5-year survival rate is 39.4% (36.4-42.4, CI 95%), with about 319 disease-related deaths every year.

For ovarian cancer patients, the chance of surviving depends both on unchangeable variables such as patient characteristics and tumor biology and on modifiable variables such as quality of treatment (surgical treatment, chemotherapy). Because treatment quality is correlated to the hospital that delivers it and because high-volume hospitals provide a better prognosis, centralizing care for ovarian cancer patients is now recommended [7,8]. In light of this recommendation, we decided to investigate the management and treatment of EOC in Emilia-Romagna. To this end, a multidisciplinary group was formed in 2009, and trained to perform clinical audits in order to identify quality indicators and to develop Region-wide workup in accordance with the principles of evidence-based medicine (EBM). The rationale was that, by setting up an oncogynecology network so as to achieve the best clinical practice, critical points would decrease or even be eliminated.

To the best of our knowledge, this is the first Italian regional audit whose aim is to assess the quality of care of EOC patients [9].

## Methods

### Study design

In 2009, 11 physicians and 3 biostatisticians whose goal was to set up an oncologic network named Rete di oncologia ginecologica della Regione Emilia-Romagna (ROGER).

A preparatory scoping audit was undertaken to identify the key issues involved in the management of EOC. Quality and standard indicators for improvement were identified after a careful revision of the literature and of the leading gynecologic societies’ guidelines (GL).

Were identified as quality and standard indicators in the diagnostic and therapeutic strategy of EOC: modality of diagnosis, washing/cytology, hysterectomy, salpingoophorectomy, omentectomy, appendectomy, peritoneal biopsy, pelvic and lumbo-aortic lymphadenectomy, peritonectomy, bowel resection, cholecystectomy, splenectomy, residual disease, chemotherapy, and hospital volume.

Clinical audit was organized in three phases. Firstly, patients treated in the period from 2007 to 2008 were retrospectively analyzed. Secondly, to evaluate further the quality of the ovarian cancer treatment, patients treated from 2009 to 2010 were also included in the analysis. Due to the failure to adhere to all 40 hospitals, it was not possible to review the data records but to evaluate all cases in the region, the analysis was based on the regional current data base. Thirdly, region-wide GL (RGL) were drafted to identify reference centers, promote centralization, promote teaching, and to ensure uniform and adequate treatment to patients treated in regional centers.

### Case ascertainment and data collection

All the patients with histological and/or cytological and/or radiological diagnosis of EOC in Emilia-Romagna hospitals were included in the audit. Patients were identified using the International Classification of Diseases, 9^th^ Revision, Clinical Modification (ICD-IX-CM) code for malignant ovarian cancer (cod. 183.0) by means of record linkage between data from hospital discharge records and from pathological database.

Each of the engaged physicians formed a team to evaluate cases from that particular hospital. Data were registered on a website designed for that purpose. The case report form included all the information necessary to evaluate the quality indicators.

Hospitals were divided in low volume (≤ 10 cases per year), medium volume (between 11 and 20 cases per year) and high volume (≥21 cases per year) and data were analysed according to the volume.

### Data handling and analysis

Data were exported electronically from the website into SAS (version 9.2; SAS Institute Inc., Cary, NC, US). The Web data entry ensured that few data were missing for clinical cases included in the analysis. Multiple comparisons were made between presenting characteristics and principal clinical outcomes of patients.

### Statistical methods

Patient data were presented as percentages and summary statistics of mean and standard deviation, where appropriate. In order to identify significant changes in clinical practice, a statistical univariate analysis was performed using, as appropriate, *χ*^2^-test, Fisher’s exact test, and Tukey’s test to evaluate the different distribution of the type of treatment, residual disease and cases volume, analysis of variance (ANOVA) test (Snedecor’s F test) and Student’s *t* test to evaluate mean age differences by treatment, and Kaplan-Meier curves and the log-rank test for survival analysis. A Cox proportional hazard regression model was used for multivariate analyses to study the effects of teaching hospitals, volume of hospitals and of age (dependent variable) on survival (undependent variable).

The differences observed were statistically significant for a *p* value less than 0.05.

SAS was used for all data analyses.

## Results

Forty hospitals that had treated at least one case of EOC in the period under study were identified. Based on ICD-IX-CM codes, 614 EOC patients treated between 2007 and 2008 were identified.

A detailed analysis was based on the review of the medical records. Between 2007 and 2008, 1,071 cases were identified throughout the Emilia Romagna Region, of these 614 (57.3%) patients were treated in hospitals participated in the audit. Two hundred and ninety-one (27.2%) medical records were included in the final analysis, whereas 223 (20.8%) cases were not included in the review because on review of the medical records, the patients were affected also other cancer or because it was not possible to be sure of the diagnosis.

The EOC patients had an age of 63.7 ± 16.5 (years ± SD). Two (20%) high volume, three (30%) medium volume and five (50%) low volume hospitals were identified.

Of 291 patients only 134 patients (46%) were treated in the high volume hospitals.

Staging was available for 248 of the 291 patients (85.2%): 50 (20.2%), 15 (6%), 131 (52.8%) and 52 (21.0%) patients were at stage I, II, III, and IV, respectively. A similar number of early stage (I-II) and advanced stage (III-IV) patients were treated in low-, medium- and high-volume hospitals (*p* = 0.23). Specifically, 28 (41.2%), 20 (29.4%) and 20 (29.4%) early stage patients were treated in high-, medium-, and low-volume hospitals, respectively. Similarly, 66 (47.8%), 46 (33.3%), and 26 (18,8%) advanced stage patients were treated in high-, medium-, and low-volume hospitals, respectively.

Two hundred and twenty-two (76.3%) patients had a histological diagnosis, 29 (9.9%) had a cytological diagnosis, and 28 (9.6%) had only a radiological diagnosis; the method of diagnosis was not reported for 12 (4.1%) patients. There were 116 patients (39.9%) with serous EOC, 32 (10.9%) patients with undifferentiated EOC, 22 (7.6%) with endometrioid EOC, 15 (5.1%) with mucinous EOC, 14 (4.8%) with clear cell EOC, and 33 (11.3%) with other hystotypes. Histotype was not reported in 59 patients (20.3%).

Survival of the 291 EOC patients according to the treatments is reported in Table 1.

**Table 1:** Survival of the 291 EOC patients treated between 2007–2008 at four years from first treatment according to the type of treatment

	**Only surgery**		**Only chemo therapy**		**Neo adjuvant chemotherapy + surgery + chemotherapy**		**Surgery + adjuvant chemotherapy**		**Supportive care**	
**Follow-up**	**N.**	**%**	**N.**	**%**	**N.**	**%**	**N.**	**%**	**N.**	**%**
Dead	16	30.2	21	46.7	9	22.5	20	16.3	30	0
Live with disease	1	1.9	6	13.3	12	30	31	25.2	0	0
Live free of disease	31	58.5	3	6.7	13	32.5	46	37.4	0	0
No data	5	9.4	15	33.3	6	15	26	21.1	0	0
**Total**	**53**	**100**	**45**	**100**	**40**	**100**	**123**	**100**	**30**	**100**

In the early stages (I-II), complete staging was not always performed and lumbo-aortic lymphadenectomy was performed only in 35% of patients (Table 2). Residual disease at first cytoreduction surgery was not reported in 30% of patients. A complete cytoreduction was obtained only in 20.1% of patients with stage III-IV (Table 2).

**Table 2:** Characteristics of the 206 EOC patients surgically treated between 2007-2008

	**Stage I**		**Stage II**		**Stage III-IV**	
**Mean age (years ± SD)**	55.6 ± 15.6			56.5 ±10.6					63.6 ± 13.0				
	**N.**	**%**	**N.**	**%**	**N.**	**%**							
**Surgical procedures**													
Washing	41	87.2	11	73.3	70	48.6							
Hysterectomy	40	85.1	15	100	96	66.7							
Annessiectomy	47	100.0	15	100	144	100							
Omentectomy	41	87.2	15	100.0	91	63.2							
Appendectomy	21	44.7	6	40.0	30	20.8							
Peritoneal biopsy	33	70.2	11	73.3	70	48.6							
Pelvic lymphadenectomy	20	42.6	10	66.7	49	34.0							
Lumbo-aortic lymphadenectomy	17	36.2	5	33.3	33	22.9							
Peritonectomy	3	6.4	1	6.7	25	17.4							
Colecistectomy	1	2.1	0	0.0	5	3.5							
Bowel resection	0	0	0	0	20	13.9							
Splenectomy	0	0	0	0	4	2.8							
**Total**	**47**		**15**		**144**								
**Residual disease**													
Not reported	17	36.2	8	53.3	37	25.7							
No residual disease	28	59.6	7	46.7	29	20.1							
<=1 cm	0	0.0	0	0.0	7	4.9							
> = 2 cm	0	0.0	0	0.0	23	16.0							
Residual disease present but the diameter is not specified	0	0.0	0	0.0	16	11.1							
Missing	2	4.3	0	0	32	22.2							
**Total**	**47**	**100**	**15**	**100**	**144**	**100**							
**Survival at four years from surgically treatment**													
Live free of disease	36	76.6	6	40.0	37	25.7							
Dead	0	0.0	2	13.3	44	30.6							
Live with disease	3	6.4	4	26.7	39	27.1							
No data	8	17.0	3	20.0	24	16.7							
**Total**	**47**	**100**	**15**	**100**	**144**	**100**							
**Type of treatment**													
Only surgery	24	46.8	-	-	8	5.6%							
Surgery plus adjuvant chemotherapy	22	51.1	14	93.3	74	51.4%							
Neo adjuvant chemotherapy plus surgery plus chemotherapy	1	2.1	1	6.7	35	24.3%							
Only chemotherapy	-	-	-	-	24	16.7%							
Supportive care	-	-	-	-	3	2.1%							
**Total**	**47**	**100**	**15**	**100**	**144**	**100**							

Only chemotherapy was administered to 45 (15.5%) patients (years ± SD, 68.8 ± 16.8), with an average of 4.8 cycles (range 1–11 cycles). Neoadjuvant chemotherapy was administered to 40 (13.7%) patients (years ± SD, 60.4 ± 14.6), with an average of 5.1 cycles (range 1–8 cycles). Adjuvant chemotherapy was administered to 123 (42.3%) patients (years ± SD, 59.9 ± 11.4), with an average of 5.6 cycles (range 1–8 cycles). Although high volume hospitals presented the higher overall survival compared with low and medium volume hospitals, these differences were not significant. Survival according to stage is reported in Table 2.

On the basis of the first set of results (2007–2008), it became clear that further evaluation would require including all patients treated in the whole region even in those hospitals which have not adhered to the audit. To do this, current regional data base was analyzed without reviewing data records. Based on ICD-IX-CM codes, 2163 EOC patients treated between 2007–2010 were thus identified (Table 3). Based on the number of treated patients, we found 2 (5%) high-volume hospitals (≥ 21 operated patients /year), 5 (12.5%) medium-volume hospitals (11–20 operated patients/year), and 33 (82.5%) low-volume hospitals (≤10 operated patients /year). Of these 40 hospitals, five (12.5%) were teaching hospitals. Median survival was 30 months.

**Table 3:** Characteristics of the 2163 EOC patients treated between 2007-2010

**Age**									**Total**
	**2007**		**2008**		**2009**		**2010**		
	**N.**	**%**	**N.**	**%**	**N.**	**%**	**N.**	**%**	**N**
**< 40 years**	33	6.3	33	6.1	35	6.2	26	4.9	**127**
**40 – 55 years**	97	18.4	98	18.0	112	19.8	97	18.4	**404**
**55 – 69 years**	172	32.7	182	33.4	182	32.2	166	31.6	**702**
**> 70 years**	224	42.6	232	42.6	237	41.9	237	45.1	**930**
**Total**	**526**	**100**	**545**	**100**	**566**	**100**	**526**	**100**	**2163**
**Treatment**	**N.**	**%**	**N.**	**%**	**N.**	**%**	**N.**	**%**	**N**
**Chemo -therapy**	46	8.7	59	10.8	56	9.9	63	12.0	**224**
**Surgery**	269	51.1	273	50.1	310	54.8	275	52.3	**1.127**
**No treatment**	211	40.1	213	39.1	200	35.3	188	35.7	**812**
**Total**	**526**	**100**	**545**	**100**	**566**	**100**	**526**	**100**	**2163**

**Figure 1:**
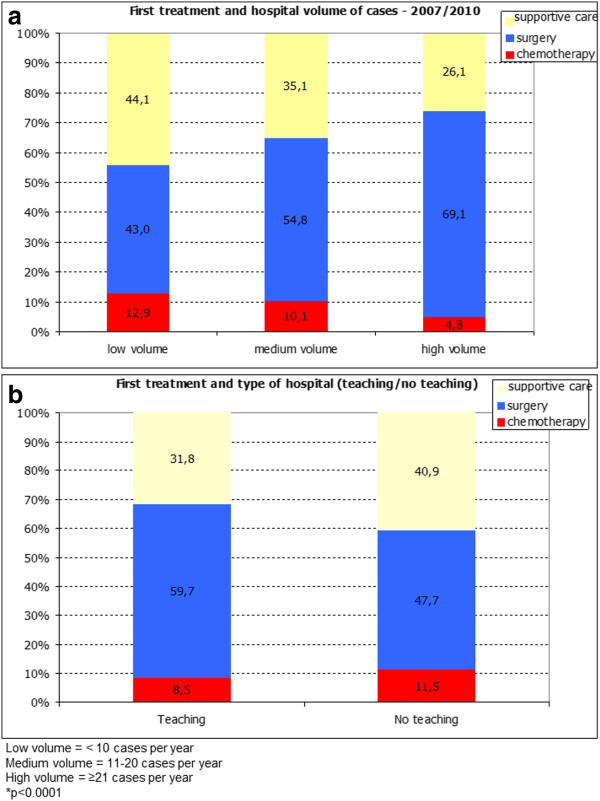
Type of first treatment according to the patients volume (a) and to the teaching (b) hospitals.

The distribution of patients according to treatment is shown in Table 3 and type of first treatment according to age is reported in Table 4. When dividing the patients according to age, it was found that the majority of patients (63.18%) aged >70 years received only supportive care. Type of first treatment according to hospital volume showed significant differences. In low-volume hospitals, significantly (*p* < 0.0001) fewer patients (43%) were surgically treated than were in medium- (54.8%) and high-volume (69.1%) hospitals and significantly (*p* < 0.0001) more patients (12.9%) were treated with chemotherapy than were in high-volume (4.8%) but similar to the number in medium-volume hospitals (10.1%). Further, significantly (*p* < 0.0001) more patients (44.1%) in low-volume hospitals received no treatment than did those in medium- (35.1%) and high-volume (26.1%) hospitals (Figure 1a).

**Table 4:** Type of first treatment according to the age of the 2163 EOC patients treated between 2007-2010

	**AGE**								**Total**
	**< 40 years**		**40 – 55 years**		**55 – 69 years**		**> 70 years**		
	**N.**	**%**	**N.**	**%**	**N.**	**%**	**N.**	**%**	**N**
**Chemotherapy**	9	4.0	38	17.0	84	37.5	93	41.5	224
**Surgery**	99	8.8	296	26.3	408	36.2	324	28.7	1.127
**No treatment**	19	2.3	70	8.6	210	25.9	513	63.2	812
**Total**	**127**	**5.9**	**404**	**18.7**	**702**	**32.5**	**930**	**43.0**	**2163**

A significantly greater number of patients treated at teaching hospitals (5 hospitals) received surgery as first treatment compared to those treated in non-teaching hospitals (35 hospitals) (59.7% *vs*. 47.7%, respectively; *p* < 0.0001). Teaching and non-teaching hospitals treated a similar number of cases with chemotherapy (8.5% *vs*. 11.5%, respectively, *p* = 0.0289) but teaching hospitals offered supportive care to a significantly lower number of cases compared with non-teaching hospitals (31.8% *vs*. 40.9% respectively, *p* < 0.0001) (Figure 1b).

**Figure 2:**
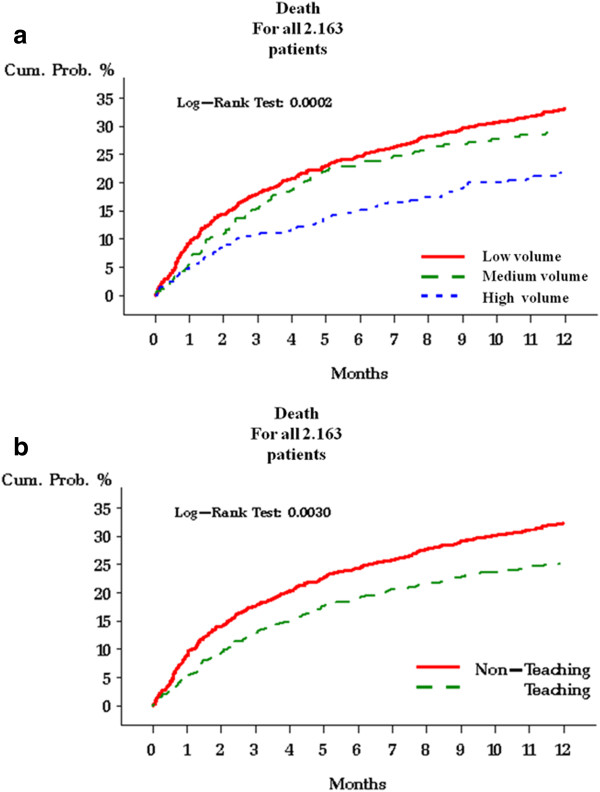
Risk of death in the 2,163 EOC patients according to the patients volume (a) and to the teaching of the hospital.

Patients treated in the high-volume hospitals presented a significantly lower risk of dying compared to patients treated in medium- and low-volume hospitals. Patients treated in teaching hospitals presented a significantly lower risk of dying compared with patients treated in non- teaching hospitals (Figure 2). Women aged ≤64 years showed a statistically higher survival compared to women aged ≥ 65 years (3.756 years and 2.273 years, respectively, *p* < 0.0001).

Hospital volume correlation with patient age was statistically significant (*p* < 0.0001): 52.4% of the women treated in high volume hospital were aged ≤ 64 years, 44.7% of those treated in medium volume hospital were aged ≤ 64 years, and 39.8% of those treated in low volume hospital were aged ≤ 64 years.

Multivariate analysis showed that lower volume hospital (compared to high) increased the risk of lower survival and that it increased slightly in not-teaching hospitals compared to teaching hospitals. Age was strongly associated with survival, with women older than 65 years having an increased risk of lower survival (HR 2.86) compared to younger women.

## Discussion

Despite several improvements in the treatment of EOC, this malignancy remains the leading cause of the death among gynecologic tumors [10].

To the best of our knowledge, the present study is the first Italian regional audit whose goal was to assess the current state of where and how treatment of EOC is provided in Emilia-Romagna Region and to determine the appropriateness of its diagnostic and treatment pathway. To this end, a regional oncology network (ROGER) was formed whose aim was to improve EOC patient outcomes by ensuring uniformity of care and treatment. The audit reviewed EOC cases treated in the period 2007–2008, discovering that of the 291 EOC patients whose charts were available for review, only 222 patients (76.3%) had a histological diagnosis, FIGO surgical staging was reported only in 206 patients (70.9%) but not all standard surgical procedures were always performed, however the 70% of the patients were at advanced FIGO stage (Table 2).

It is unacceptable that patients for whom no reliable histological diagnosis and staging is available are treated without proven indication to the chemotherapy. Yet our audit indicated that this may have occurred.

While our audit brought to light some very troubling and serious lapses in diagnosis and treatment, similar scenarios reported in the literature show that the Emilia-Romagna region is not alone. To this regard, in 2003, the Advanced Chemotherapy in Ovarian Neoplasm (ACTION) trial re-ported that inadequate staging affected the outcome of the trial (only 34% of the FIGO stage I-IIA patients were staged), despite the fact that strict staging guidelines had been given [9-14]. Probably the omission of staging was due to the lack of surgical expertise in fact it occurred more frequently at institutions that had enrolled fewer than five patients. However, only 37% of patients even from institutions entering >20 patients were completely staged. Similar findings were reported in the Surveillance, Epidemiology and End Results Program study [15-17].

Today, the aim of surgery in ovarian cancer is the complete resection of the tumor [18-20]. Our audit showed that only 20.1% of advanced-stage patients underwent complete cytoreduction, with a further 30.1% of the patients for whom this parameter was not reported, despite it is the most important prognostic factor and quality indicator of the surgery [21]. Recent studies have confirmed that surgical outcome depends on the surgeon and hospital-related factors rather than on the spreading pattern of the tumor [17,22].

A greater diversity of treatment was also observed in the administration of neoadjuvant chemotherapy; the median number of cycles administered to the patients was 5.1 (range 1–8 cycles) despite the fact that 3 cycles of chemotherapy are usually suggested; more would delay cytoreductive surgery, resulting in potentially detrimental effects on the survival in advanced EOC [12].

Given these critical findings for 2007–2008, we felt that a larger-scale evaluation was opportune and thus extended the audit to include all the EOC patients treated in the whole region 2009–2010 as well.

Considering all the EOC patients treated from 2007 to 2010, only the 26.8% of EOC patients underwent surgery in the high-volume hospitals. In the 2 high-volume hospitals 69.1% of the patients underwent surgery as primary treatment; surgery represented primary treatment also in the 59.7% of the patients of the five teaching hospitals. These findings are not surprising, given that in the Regional Healthcare System of Emilia Romagna there is no centralization for EOC patients.

Above all, in line with the literature, regardless of stage, the patients treated in the high-volume hospitals presented a significantly lower risk of death compared with patients treated in medium- and low-volume hospitals regardless of patient age and structure (teaching or not teaching). Patients treated in teaching hospitals presented a significantly lower risk of dying compared with patients treated in non-teaching hospitals. This finding emerged despite the fact that the majority (but not statistically significant) of advanced stages were operated in high-volume hospitals.

However, it must be emphasized that the patients treated in the medium and low volume hospitals were progressively and significantly older than those treated in the high volume hospitals and that the age proved to be strongly related to lower survival in multivariate analysis. The older patients may have had a lower survival for several reasons, including the coexistence of other pre-existing diseases or the surgeon’s propensity to limit surgery in elderly patients with comorbidities. These findings are in accordance with the literature, where it is reported that elderly women with EOC are less likely to be centralized and to undergo surgery by an oncogynecologist [23].

However, results from the current regional data base are weakened because they were not on reviewed data records, so these data should not to be over interpret, but they represent a preliminary analysis.

Over the last decade, several studies have reported the importance of centralizing care of ovarian cancer patients to guarantee the assistance of specialized gynecologists, thereby improving prognosis/ survival [7-11]. It is discouraging to see that our regional findings are not much better than those reported in a national Italian audit performed in 1988, after national guidelines were provided by the National Research Council . That analysis showed serious deficiencies in diagnostic and staging procedures and information on grading and residual tumour was available only in 30% and 45% of cases, respectively, and only 10% of the patients had random biopsies as part of their surgical staging [24].

The differences between hospitals in terms of treatments provided and the differences between the treatments provided and the gold standard led the multidisciplinary group to draft standardized Regional GL (RGL) to identify reference centers, to promote centralization, and to ensure uniform and adequate treatment to patients treated in regional centers [25].

## Conclusion

Despite we reviewed only the 47.4% of the EOC cases treated in period 2007–2008, our audit has played a pivotal role in assessing the quality of regional assistance and in highlighting critical points such as the inconsistent approach to care and the failure to apply international GL. This audit has allowed us to perform a preliminary analysis of how, where, when, and by whom EOC is treated in regional hospitals. These results are a wake-up call tense that should push all professionals in the region to take part in a future audit for a more rigorous effort for a more accurate assessment of all patients treated in the region.

## Abbreviations

EOC: Epithelial ovarian cancer; ROGER: Rete di oncologia ginecologica della Regione Emilia-Romagna; US: United States; FIGO: International Federation of Gynecology and Obstetrics; GL: Guidelines; ICD-IX-CM: Clinical Modification code; ANOVA: Analysis of variance; SD: Standard Deviation; HR: Hodds Ratio; ACTION: Advanced Chemotherapy in Ovarian Neoplasm.

## Competing interests

The authors declare that they have no competing interests.

## Authors’ contributions

**VDM** conceived the manuscript, performed operations, collected data, wrote manuscript. **MA** conceived the regional audit, performed operations, collected data, wrote manuscript. **PDI** conceived the regional audit, collected data, performed operations. **DP** took part to the regional audit, collected data, performed operations. **GC** took part to the regional audit, collected data, performed operations. **ML** took part to the regional audit, collected data, performed chemotherapies. **GC** took part to the regional audit, collected data, performed operations. **AV** took part to the regional audit, collected data, performed operations. **BL** took part to the regional audit, collected data, performed chemotherapies. **AA** took part to the regional audit, collected data, performed operations. **FR** took part to the regional audit, collected data, performed operations. **RM** took part to the regional audit, collected data, performed operations. **RDP** conceived data sheet, performed statistical analysis. **CV** performed statistical analysis. **LMBB** performed statistical analysis. **DF** performed multivariate analysis. **GBLS** wrote and revised the manuscript. All authors read and approved the final manuscript.
